# Exploring Molecular Insights of Cereal Peptidic Antioxidants in Metabolic Syndrome Prevention

**DOI:** 10.3390/antiox10040518

**Published:** 2021-03-26

**Authors:** Fred Kwame Ofosu, Dylis-Judith Fafa Mensah, Eric Banan-Mwine Daliri, Deog-Hwan Oh

**Affiliations:** 1 Department of Food Science and Biotechnology, College of Agriculture and Life Sciences, Kangwon National University, Chuncheon 24341, Gangwon-do, Korea; ofosufk17@kangwon.ac.kr (F.K.O.); ericdaliri@yahoo.com (E.B.-M.D.); 2 Department of Family and Consumer Sciences, College of Applied Science and Technology, Illinois State University, Normal, IL 61761, USA; dylisjudithmensah@gmail.com

**Keywords:** cereal grains, antioxidant peptides, oxidative stress, metabolic syndrome, functional food

## Abstract

The prevalence of metabolic syndrome (MetS) is presently an alarming public health problem globally. Oxidative stress has been postulated to be strongly correlated with MetS, such as type 2 diabetes, obesity, hypertension, cardiovascular diseases, and certain cancers. Cereals are important staple foods which account for a huge proportion of the human diet. However, owing to recent growing demand and the search for natural antioxidants for the prevention and management of MetS, cereal peptides have gained increasing attention for developing functional ingredients or foods with substantial antioxidant properties. This review explores the current production techniques for cereal peptidic antioxidants and their potential mechanism of action in the prevention and management of MetS.

## 1. Oxidative Stress

The term “oxidative stress” was initially conceptualized about three (3) decades ago [1,2]. Since then, the term has evolved remarkably. The inception of oxidative stress finds its roots in early publications of Seyle (loc. cit), which were concerned with the toxicity of oxygen linked with aging, bodily responses and processes associated with oxygen radicals, the concept of the physiology of the mitochondria and research on its aging, as well as work on variances of redox reactions in living organisms [1]. Table 1 shows several definitions proposed by different scientists over the years. Consequently, oxidative stress is denoted by an imbalance between oxidant and antioxidant species, such that oxidant species weigh more, which results in the excessive release of free radicals or reactive oxygen species (ROS) and causes cellular and molecular disruption, as well as a negative influence on redox signaling [3,4,5]. Oxidative stress occurs when antioxidant defenses are impaired or are not strong enough to overpower the production of reactive oxygen species (ROS) [3,4,6]. The consequences of oxidative stress may be progressive and often dire. The two underlying components in oxidative stress, as evidenced by the definition above, are prooxidant and antioxidant species. It is their non-homeostatic co-existence that results in oxidative stress. To prevent excess ROS production in mammalian cells, antioxidant molecules and antioxidant enzymes act as defense systems. There are many prooxidant species and antioxidant species. In cells, glutathione (GSH) is the most abundant and important non-protein antioxidant molecule. Antioxidants are those substances that counteract the harmful effects of oxidants. They are usually produced in insufficient quantities by the body; therefore, they need to be supplemented frequently from external sources, typically food sources [7]. Examples of antioxidants are vitamins A, C, E, carotenoids (including beta-carotene and astaxanthins), and polyphenols (such as flavonoids, isoflavones, anthocyanins, chlorogenic, and catechins) [7,8,9]. Examples of prooxidants, particularly the reactive species, include reactive oxygen species (ROS) [6,10], reactive sulfur species (RSS) [11], reactive electrophile species (RES) [12], reactive carbonyl species (RCS) [13,14], reactive nitrogen species (RNS) [15,16,17], and reactive halogen species (RHS) [10,15,18]. ROS, RHS, and RNS are toxic oxidants that cause damage to DNA, RNA, lipids, and phagocytosed pathogen proteins, especially during inflammation [10]. According to Yang et al. [19], ROS, RHS, and RNS cause apoptosis by directly oxidizing protein, lipid, and DNA signaling pathways, which increases the risk of CVD, specifically atherosclerosis. RSS are molecules produced from sequential one-electron oxidations (loss of electrons in a chemical reaction) of hydrogen sulfide, thus forming thiyl, hydrogen persulfide, and the persulfide “supersulfide” radicals, before terminating in elemental sulfur [11]. There are resemblances between ROS and RSS, and they are sometimes misconstrued and used interchangeably. However, RSS have more effectiveness, reactivity, signaling, and versatility potential compared to ROS. They can also be accumulated and reused [11]. RES have a wide range of functionality with overlying chemical reactivity, which sometimes makes the study of biological RES challenging. Biologically, they range in different shapes and forms. RES involved in cell signaling may even rise higher when they sense the human body is stressed. RHS control antioxidant response, cell growth, DNA damage development, aging, cellular homeostasis events, such as apoptosis, and immune response [12].

**Figure 1:**
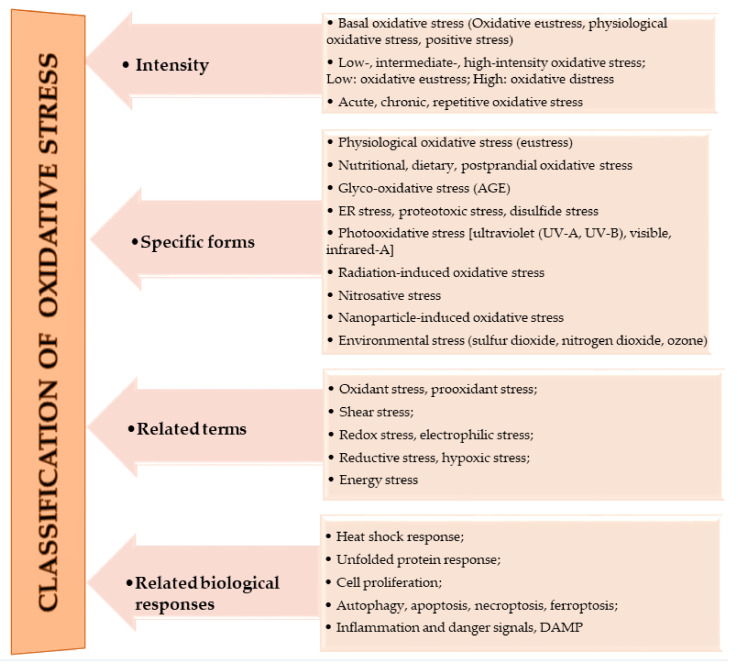
Classifications of oxidative stress adapted with permission from Sies et al. [4]. Copyright 2017 Annual Reviews, Inc.

The oxidation of macromolecules, such as carbohydrates, lipids, and amino acids, produces many reactive carbonyl species (RCS). RCS can react and cause changes to the surface composition of proteins, nucleic acids and amino phospholipids, and therefore serve as agents of cell destruction and gene mutation. Furthermore, interaction of RCS with biological samples results in many chemical products that have diverse negative effects on human health [13]. RCS are shown to be involved in ROS signaling, and are typically classified under RES [14]. Electrophilic and nucleophilic reactions are some of the most common covalent bond formations that are found in many chemical–cellular reactions. The electrophilic nature of carbonyl compounds has a high affinity for nucleophilic cellular constituents, thus making it possible for easy accessibility of the cells by RCS to render its physiological effects [13,14]. In the form of free radicals, such as nitric oxide and other nitric oxide-derived species (organic species, such as 3-nitrotyrosine (3-NT) and S-nitrosothiols (RS–NO), and inorganic species, such as nitrite [15]), RNS are involved in a variety of biological activities, and their detection and quantification are technically difficult [15,16]. However, there is evidence that, when RNS is produced in excess, it disrupts protein synthesis in the body, which harms mitochondrial metabolism dynamics and mitophagy in the nervous system [17]. RHS, especially those with chlorine, bromine, and iodine (thus, HOX with X=Cl, Br or I) induce injuries to DNA, RNA, lipids, and proteins cells [10,15]. They render the immune system’s defense useless when they are released into the body in high quantities by reversing the action of phagocytes (including neutrophils, monocytes, macrophages, mast cells, dendritic cells, osteoclasts, and eosinophils) that capture pathogens, and, thus, instigate disease or infection pathogenesis [10]. When RHS interreact with extracellular myeloperoxidase (MPO) produced by neutrophils to provide immune system defenses against pathogens, it results in tissue damage, cellular damage, and inflammatory reactions that facilitate the occurrence of a variety of diseases, particularly CVD (atherosclerosis), obesity, T2DM, and, ultimately, metabolic syndrome (MetS) [10,18]. Owing to the vast quantities and differences in prooxidant and antioxidant species, the best way to understand oxidative stress is through a classification system. According to Sies et al. [4], oxidative stress can be classified according to its intensity (basal, low, intermediate, and high), specific forms, related terms, and associated biological responses (Figure 1). A further assessment by Lushchak [20] establishes that imbalance could also result from one or a combination of the following: elevated levels of endogenous and exogenous compounds entering autoxidation, coupled with ROS production; depleted stores of low molecular mass antioxidants; deactivated antioxidant enzymes; and reduced production of antioxidant enzymes and low molecular mass antioxidants. These determine the three types of oxidative stress—acute, chronic, and quasi-stationery [20,21,22,23] (Table 2).

## 2. Metabolic Syndrome (MetS)

Insulin resistant syndrome, MetS, or dysmetabolic syndrome are other terms used to denote metabolic syndrome. MetS is a health condition characterized by the summative complexities of three or more of the following risk factors: high blood pressure, abdominal obesity, elevated triglyceride (TG) levels, low high-density lipoprotein (HDL) levels, and high fasting levels of blood sugar that instigate the potential for cardiovascular diseases (CVDs) and type 2 diabetes mellitus (T2DM) [28,29,30]. Typically, low high-density lipoprotein (HDL) levels are less than 40 mg/dL in men, or less than 50 mg/dL in women; elevated TG is 150 mg/dL of blood or higher; elevated fasting glucose is l00 mg/dL or higher; and high blood pressure is indicated by systolic levels of 130 mmHg or higher and/or diastolic levels of 85 mmHg or higher [31]. Research purports a positive correlation between increases in age and obesity, and the prevalence of MetS [32,33]. Sigit et al. [34] argue that MetS is sex and population specific. Current trends suggest Asian populations are showing, relatively, the highest prevalence (an estimate of 39.9 ± 0.7%) [34,35]; about one-third of US adults (thus, 34.3 ± 0.8% of all adults and 50% of those aged 60 years or older) have MetS [33,36]; and about 29.2 ± 0.7% in European populations [34,35,36]. Comparatively, the prevalence is very minimal in populations in Africa; however, diabetes, one of the pre-indicators of MetS, is expected to have huge spikes in sub-Saharan Africa, the Middle East, and North Africa (141% and 104%, respectively) in the next 25 years [30]. It is worth noting that sex and populations showed differing prevalence and contributing risk factors of MetS. For instance, in relation to sex, the incidence of MetS was higher in Indonesian women than in Indonesian men, but higher in Dutch men than in Dutch women [34]; in relation to contributing risk factors, Asian–Chinese men recorded higher TG and FBS levels than European men. Again, Chinese women recorded higher glucose levels than European women [34,37]; abdominal obesity was higher in Dutch women than in Indonesian women [34].

### The Link: Oxidative Stress and Metabolic Syndrome

**Figure 2:**
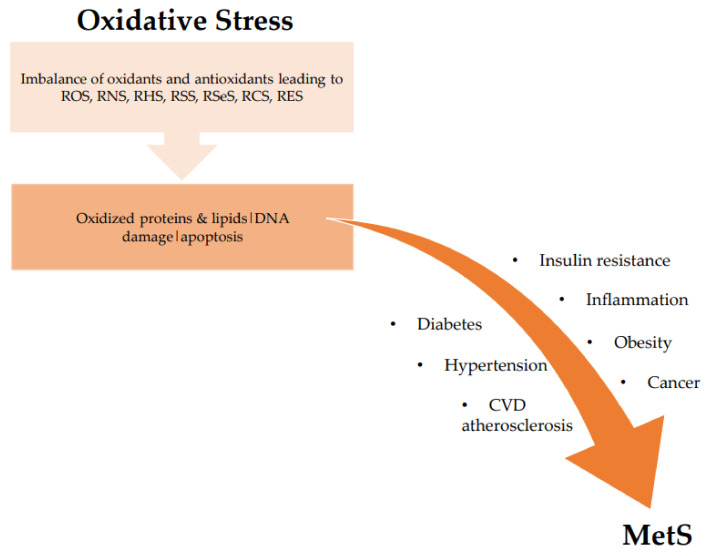
Association between oxidative stress and MetS. ROS: reactive oxygen species; RNS: reactive nitrogen species; RHS: reactive halogen species; RSS: reactive sulfur species; RSeS: reactive selenium species; RCS: reactive carbonyl species; RES: reactive electrophile species; CVD: cardiovascular diseases.

There is compelling evidence of the association between oxidative stress and MetS from both human and animal studies [38,39,40,41]. Oxidative stress correlates with increased BMI, increased adiposity, and high blood pressure, which are all components of the risk factors of MetS. Particularly in obesity-related MetS, elevated ROS production targets adipose tissue and rapidly incites fat accumulation, thereby contributing to atherosclerosis, among other vascular medical conditions [40,41,42]. Oxidative stress reduces the number of helpful proteins, such as adiponectin (involved in regulating glucose levels and fatty acid breakdown); as adiponectin decreases, systemic oxidative stress levels increase. Oxidative stress associated with increased adiposity mediates the development of MetS [38,42,43]. There are two main mechanisms by which this occurs: first, through increased oxidative stress in accumulated fat, resulting in uncontrolled adipocytokines production; and second, the selective rise in reactive species production in accumulated fat, resulting in high systemic oxidative stress [38,39,40,41]. Oxidative stress impairs pancreatic beta cells’ ability to secrete insulin, affecting glucose transport in muscle and adipocytes which relates to the development of hypertension and diabetes [38,42]. The association between oxidative stress and MetS is shown in Figure 2.

## 3. Antioxidative Peptides

Bioactive peptides (BP) are short protein fragments obtained from food proteins that can provide beneficial effects to the human body system due to their unique health-promoting properties [44]. BP differ from protein by their small molecular weight, formed by short sequences of amino acids that are linked together by amide or peptide bonds [45]. BP usually contain between 3 and 20 amino acid residues, and remain inactive while the sequences are kept within the parent protein. They are activated by the peptidases that incite enzymatic hydrolysis during food processing and/or during gastrointestinal (GI) digestion [46]. During digestion, BP must cross the gastrointestinal barrier and survive enzyme degradation in order to have a positive health effect [47]. Depending on the sequence of the amino acid sequence, BP render many functional, physiological, and therapeutic properties, including antihypertensive [48], antioxidant [48,49], anti-obesity [50], antifungal, antiviral, immunomodulatory [51], antimicrobial [51,52], anticoagulant [53], anti-inflammatory [54], antidiabetic, iron- and calcium-binding activities [55], antiproliferative [56] and chelating effects [57]. They also account for the taste of food and the inhibition of enzymes involved in developing diseases. Functional foods obtained from animal and plant sources have been shown to contain high-value BP with primarily antioxidant properties. A few examples of these animal and plant sources of BP include seafood [58,59], egg [55], goat milk [60], bovine [61], rice bran [62], soybean [63] and cauliflower byproducts [64].

While still emerging, the applications of food-derived antioxidants are varied and diverse. There is evidence of their utilization in food systems [65], nutrition and nutraceuticals, additive development, therapeutics [66], and food preservation [67]. In animal-derived antioxidant peptides, such as in milk, they have been used successfully as free radical scavengers [68]. Plant-derived antioxidants are used for taste enhancement and as health fortification agents [69], such as liver protectants [70], memory enhancers [71], and supplements to promote healthy cells [72,73]. In therapeutics, plant-derived antioxidant peptides are effective in treating blood disorders [74], hypertension [75,76] and malaria [77,78]. Among the plants’ sources, cereals have been extensively studied and found to exert substantial antioxidant potential to alleviate oxidative stress. Cereals account for a huge proportion of the human diet, and the presence of mono- or multifunctional peptides contributes to an increase in their food protein quality for daily consumption [79]. The amino acid composition, sequence and molecular weight are key factors that contribute to the antioxidant activities of peptides [80].

## 4. Cereals Historical Significance and Assortments

History has it that the word “cereals” is traced to Ceres, the Roman goddess who symbolizes the power to produce food—agriculture and cultivation, fertility, abundance, and nurturance. In fact, ancient Romans are credited for a sculpture of Ceres which sits on the arch of the Chicago Board of Trade assembling—a significant place for agricultural products’ marketing. The old Romans are generally recognized as the pioneers of the cereal industry. They were the first to introduce cereal processing (thus, crushing of grain and utilizing flour to make refined white bread) [81]. Cereals refer to an edible seed of the grass which comprises a germ (the innermost core, rich in fats and vitamins), the endosperm (the middle layer, rich in carbohydrates and some traces of protein and vitamins), which is housed by bran (the outermost layer of fiber and B-vitamins) [82]. They serve as highly dense carbohydrate foods for many populations worldwide [83,84]. They are edible seeds from members of the grass family Gramineae or Poaccac [49]. They are also referred to as grains; however, grains compose both the fruit (kernel or seed) and the grass [81]. Maize, wheat, barley, rice, oat, millet, sorghum, and rye are amongst the most famous types of cereals; however, wheat takes the lead globally [49]. Although the cereals mentioned above belong to the same family, they each have a unique genus, as shown in Table 3.

### 4.1. Cereals as Sources of Antioxidant Peptides

#### 4.1.1. Oats

Oats contain prolamins known as avenins [82]. There are two types of bioactive peptide properties in oats—antioxidant and anti-inflammatory. High potent antioxidants are typically associated with peptides containing 2–20 amino acids with a molecular weight of less than 3 kDa, and with the presence of hydrophobic amino acids, such as proline (P), valine (V), tryptophan (W), and phenylalanine (F). Amino acids, such as valine (V) and leucine (L), have also been reported to provide antioxidant properties when found at the N-terminal of a peptide. Oats are rich in avenanthramide, a polyphenol whose components are characterized by a pseudo-peptide bond known to possess both antioxidant and anti-inflammatory activities [85]. Avenanthramides decrease inflammation-related cytokine production by inhibiting specific nuclear factor activities. Certain species of oats have special anti-inflammatory potency towards autoimmune diseases, such as celiac disease [56]. Oats have a high oxygen radical absorbance capacity, (ORAC) related to the elevation in antioxidant enzymes’ action, and significantly decrease the occurrence of oxidative stress and other harmful specific nuclear factor activity protein levels.

#### 4.1.2. Wheat

Gluten in wheat incites inflammation responses among some minute allergen-stricken populations, particularly those with celiac [86,87]. Prolamins in wheat are called gliadins [82]. Wheat antioxidant peptides reduce the occurrence of tumor necrosis [56,88]. Several peptides or protein hydrolysates, with antioxidative properties obtained from wheat bran, germ and gluten, are shown in Table 4, Table 5, Table 6 and Table 7.

#### 4.1.3. Rice

Rice has various benefits, right from the germ through to its outermost layer (bran), which is usually lost through the series of food processing [62]. Rice bran is rich in albumin hydrolases, which are potent for combating hypertension, diabetes, and oxidation in cells through antioxidant and α-glucosidase, as well as angiotensin-converting enzyme (ACE)-inhibitory activities [62,89]. Antioxidants and anti-inflammatory properties, mainly in black rice, have been linked to the lowering of inflammation markers, such as high–sensitive C-reactive proteins (CRP) and risks of cardiovascular diseases (specifically, coronary heart disease and atherosclerosis) [56,62,89]. Examples of different rice peptides and protein hydrolysates demonstrating potent antioxidant activities are shown in Table 4, Table 5, Table 6 and Table 7.

#### 4.1.4. Barley

Barley is a crop used for humans and livestock, and has recently drawn attention as a medicinal grain. Prolamins constitute 30–50% of barley’s total proteins and are called hordeins [49,82]. Research studies over the past decade have shown the biological activities of barley [56]. Flavourzyme and alcalase hordein hydrolysates exhibited superior DPPH free radical scavenging activity (70%) at 0.5 mg/mL, Fe^2+^-chelating ability (73%) at 1 mg/mL, and superoxide radical scavenging capacity [90]. The potential of barley to function as an anti-inflammatory and antioxidant has been tested in both human and animal studies. In the beverage industry, where indignant alcohol consumption has been positively correlated to induce excessive production of ROS, resulting in liver damage, fermented barley is a healthier choice. Fermented barley extracts are linked to improving antioxidant activities by assuaging alanine aminotransferase and aspartate aminotransferase levels [56,91,92]. Additionally, fermented barley extracts were shown to suppress IL-1, IL-6, and tumor necrosis factors in lipopolysaccharide (LPS)-induced inflammation in rats, thus reiterating the anti-inflammatory activities of barley extracts [91]. The fundamental principle of the anti-inflammatory effects of fermented barley extracts was related to the upregulation of antioxidant enzyme activities and the downregulation of oxidative stress. From the aforementioned, it is evident barley has potential benefits in fighting inflammation-related diseases [56,92].

#### 4.1.5. Rye

Rye is typically considered one of the minor grains. Prolamins, the major storage proteins in rye, are referred to as secalins [49,82,85]. Rye resembles wheat and barley, and is predominantly cultivated in Europe. Rye is famous for food products, such as whiskey and bread [56]. Four tripeptides (CQV, QCA, QCV and QVC) derived from rye secalin demonstrated hydroxyl radical scavenging and chromium-VI (Cr(VI) reducing activities [93]. Rye sourdough fermentation with lactic acid bacteria resulted in the synthesis of antioxidant peptides PAEMVAAALDR, KVALMSAGSMH, LCPVHRAADL, and RLSLPAGAPVTVAVSP. These peptides showed high DPPH radical scavenging activity, linoleic acid autoxidation inhibition, and cytoprotection on H_2_O_2_-induced mouse fibroblasts oxidative damage [94].

#### 4.1.6. Maize

Like all cereals, maize (also known as corn) seed basically consists of an embryo, an endosperm, and bran; the starch grains are found in the endosperm (which contains mostly amylopectin and amylose, followed by proteins) [83]. The most abundant proteins are albumins, globulins, prolamins, and glutelins found in endosperm and germ, with glutamic acid being the most abundant amino acid [95]. Maize has been found to provide bioactive peptides that are essential for nutraceutical functions [72]. The enzymatic hydrolysis of the maize kernel produces various bioactive peptides found in maize. Recently, Ortiz-Martinez et al. [82] reported on the anticancer effects of corn peptides in HepG2 cells. In other words, some maize bioactive peptides have shown profound antioxidative effects by the presence of specific amino acids, such as proline (P), tyrosine (Y), histidine (H), phenylalanine (F), lysine (K), and histidine (H), that are very strong in reducing ROS [96]. Tang and Zhuang [95] found low molecular weight peptides (less than 5kDa) to be powerful antioxidants. These small peptides showed hydroxyl radical and free radical scavenging activity, inhibition of lipid peroxidation, and ion chelating capacity properties. In corn gluten meal, CSQAPLA demonstrated antioxidative activities by scavenging superoxide anion and hydroxyl radicals [72,96]. Some examples of corn antioxidant peptides are shown in Table 4, Table 5, Table 6 and Table 7.

#### 4.1.7. Millet

Millets, numerous small seeds, are cultivated in warm countries as cereal crops or grains, and are used to produce flour and alcoholic drinks for fodder and human consumption. Millets are classified into major and minor types. The major type includes pearl millet (*pennisetum glaucum*), proso, or white millet (*panicum miliaceum*), foxtail millet (*setaria italic*), and finger millet (*eleusine coracana*) [97]. Whereas, barnyard millet (*echinochloa spp*.), little millet (*panicum sumatrense*), guinea millet (*brachiaria deflexa*), browntop millet (*urochloa ramose*), kodo millet (*paspalum scrobiculatum*), and sorghum (sorghum spp.) constitute minor millets [98]. A typical millet protein has been shown to contain albumin, globulin, cross-linked prolamins and glutelin, and some essential amino acids, such as cysteine (C) and methionine (M) [99]. Recently, the use of millet as nutraceuticals and therapeutic agents is on the rise because of its high content of health-promoting BP, which have promising antioxidants, anti-inflammatory, antihypertensive, antidiabetic, and anticancer effects [97]. Hu et al. [100] reportedly identified seven peptides from foxtail millets (predominantly EDDQMDPMAK and QNWDFCEAWEPCF) with high anti-inflammatory properties responsible for reducing ROS activity and increasing levels of glutathione and superoxide dismutase activity in Caco-2 cells. Recently, identified peptide sequences VAITLTMK and VSKSVLVK, from lower millets, and TSSSLNM, VRGGLTR and STTVGLGISMRSASVR, from finger millets, demonstrated strong antioxidative properties [101,102].

#### 4.1.8. Sorghum

Globally, sorghum ranks as the fifth most cultivated cereal [103]. Due to its high adaptability to high temperatures and drought, sorghum is very commonly cultivated in many suburbs of Africa, Asia, and other semi-desert climate areas of the world [102,104,105]. Sorghum is gluten-free. α-kafirin is the main storage protein in sorghum [104]. However, proteins in sorghum are relatively lower in quality compared to other cereals [103]. A study by Agrawal et al. [102] on green tender sorghum varieties concluded that hydrolyzed proteins in sorghum resulted in significant antioxidant performance by scavenging DPPH, 2,2′-azinobis-3-ethyl-benzothiazoline-6-sulphonate (ABTS) radicals and promoting metal chelating, and could be applied in the development of nutraceuticals. Though studies are still emerging on the health benefits specific to antioxidative peptides, many varieties of sorghum are reported to be rich in 3-deoxyanthocyanidins, tannins, and polycosanols, which are effective in solidifying gut microbiota roles against obesity, dyslipidemia, oxidative stress, inflammation, diabetes, hypertension, and some types of cancers [102,104,106,107]. Needless to say, these are potentially anti-inflammatory, cholesterol-lowering, antioxidant characteristics typically evident in BP. Table 4, Table 5 and Table 6 shows examples of sorghum and millet antioxidant peptides.

## 5. Production Strategies for Antioxidant Peptides

**Figure 3:**
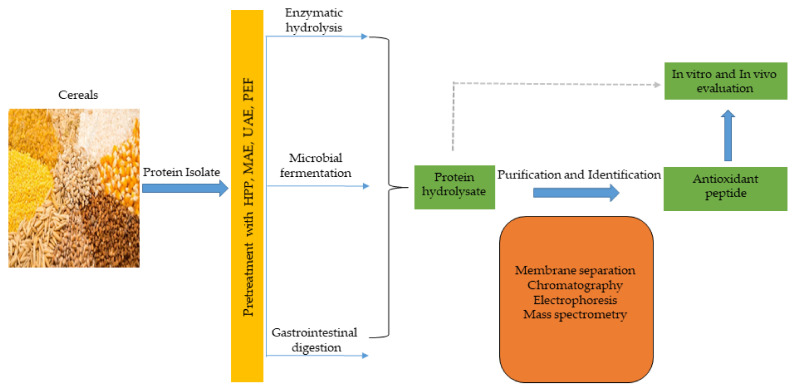
Cereal peptidic antioxidants production strategies.

Antioxidant peptides, like other functional peptides, are encrypted within the sequence of parent protein molecules and can be liberated through proteolytic enzyme hydrolysis, gastrointestinal digestion, or microbial fermentation. However, antioxidant peptides have been produced largely using enzymatic hydrolysis. In recent times, innovative and efficient food processing techniques, such as high hydrostatic pressure (HHP), microwave assistance (MA), ultrasonic assistance, pulsed electric field (PEF), and subcritical water have been employed in combination, or simultaneously, with conventional (classical) methods to improve yields and produce mono- or multifunctional peptides with lesser cost and time. These emerging technologies have been comprehensively discussed in recent reviews [48,134]. The production of antioxidant peptides from cereals and other plant sources is shown in Figure 3, and involves protein isolate pretreatment with novel food processing technologies (HHP, MA, PEF etc), followed by protease hydrolysis to obtain protein hydrolysate. Further purification steps using membrane separation, chromatographic methods, including ion–exchange chromatography (IEC), gel permeation chromatography (GPC), and reversed–phase high performance liquid chromatography (RP-HPLC), are performed to obtain antioxidant peptides, which are later identified by mass spectrometry (electrospray and tandem) and their activity is evaluated in vitro and in vivo tests [66,135].

### 5.1. Conventional Approach

#### 5.1.1. Enzymatic Hydrolysis

Hydrolysis of food proteins to produce multi- or monofunctional peptides has been mainly conducted using enzymes. Bioactive peptides have gained much attention over the years and there has recently been particular interest in antioxidative peptides due to their potential multifunctional activities in the prevention and management of several diseases. Exogenous enzymes, specifically those from microbial sources (Alcalase, Flavourzyme and Protamex^TM^), animal sources (pepsin and trypsin), and plant sources (e.g., papain), have extensively been used to produce antioxidative peptides [136]. Compared with the preparation methods of other peptides, the enzymatic hydrolysis method has the advantages of absence of organic solvents or toxic chemicals, easy reaction control, good repeatability, low cost, and low energy consumption. Therefore, it is the most widely preferred method for industrial production. However, decreasing hydrolysis time, the amount of enzyme, and improving the yield and bioactivity of peptides remain main challenges to be circumvented [137]. Consequently, innovative and emerging technologies, such as high hydrostatic pressure (HHP), microwave, and pulsed electric field, are being used to induce protein unfolding and activation of enzymes, thereby improving the efficiency of enzyme hydrolysis and generating peptide fractions with strong antioxidant activities [134]. The application of emerging technologies to produce high value BP will be discussed in detail later in this review.

The generation of protein hydrolysates and/or peptides with desirable functional properties is mainly affected by several factors, such as the types of enzyme and hydrolysis conditions (temperature, pH, the ratio of enzyme to substrate, and time), among others. It is worth noting that different types of enzymes can specifically cleave different positions of the peptide. For example, pepsin digestion increases hydrophobicity by cleaving peptide bonds between hydrophobic amino acids (preferably aromatic amino acids, including Phe, Trp, and Tyr), resulting in an appropriate reaction of rice bran protein hydrolysates (RBPH) with DPPH free radicals. Nonetheless, a reduction in this ability was observed due to the action of trypsin, which further selectively cleaves basic amino acids [120]. Thus, the type of peptidase is crucial in determining the size, composition, and amino acid sequence of the peptides, and thus affects antioxidant activity [138]. Peptidase is mainly divided into animal, plant, and microbial proteases. In general, pepsin and trypsin are the most widely used animal proteases. Suetsuna and Chen [111] reported that the two peptides (Leu-Gln-Pro-Gly-Gln-Gly-Gln-Gln-Gly and Ala-Gln-Ile-Pro-Gln-Gln) obtained from wheat gluten protein by using pepsin have good antioxidant capacities. The digestion of food proteins by digestive enzymes, or enzymes produced by microbial flora residing in the gut, offers another opportunity to produce peptides with antioxidative activities directly or indirectly in the gut, or through cell signaling pathways or their ability to enter blood circulation by crossing epithelial cell membrane to target sites [136,139]. Therefore, the stability or resistance of food-derived antioxidative peptides to GI breakdown or modification is an essential factor to be considered to ensure they exert their beneficial health properties.

#### 5.1.2. Microbial Fermentation

Microorganisms degrade food components through their complex enzyme systems. Bacteria belonging to the *Lactobacillus* genus are largely used for microbial fermentation to produce foods with improved nutritional properties and functional health benefits. Lactic acid bacteria (LAB) have a long history of use in fermented foods, and are generally regarded as safe (GRAS) [140,141]. An extracellular proteinase, a transport system specific for small peptides, and a multitude of intracellular specific, generic, endo-, and eso-peptidases constitute the proteolytic system of LAB [142]. Cereals are key components of daily diets in most parts of the world and, hence, their fermentation generates peptides with great biological importance. Antioxidant properties of cereals have been improved via the use of microbial bioprocessing and endogenous proteolytic enzymes to release antioxidant peptides and phenolic compounds from plant matrix. Antioxidant peptides have been produced and isolated from the sourdough fermentation of cereals [143]. Different strains have been reported to possess different proteolytic and peptidase activities on cereal proteins to release several peptides [141,143,144]. Galli et al. [143] revealed that different lactobacilli showed specific proteolytic and peptidase activity in wheat sourdough, which resulted in the production of low molecular weight peptides that exerted antioxidant and anti-inflammatory activities on RAW 264.7 murine macrophage, murine H-end endothelium cells, and human intestinal Caco-2 cells.

The effects of cooking on the anti-inflammatory and antioxidant properties of wheat sourdoughs and bread produced by three Lactobacilli strains (*L. farciminis* H3 and A11 and *L. sanfranciscensis* I4) were assessed by Luti et al. [145]. Peptides from dough and bread were found to suppress the NFkB pathway and, also, to reduce intracellular ROS levels. Biological activities were retained after cooking, despite differences in amino acid compositions and sequences between dough and bread peptides. Wheat germ fermented with *Lactobacillus plantarum* DY-1 exhibited a hydroxyl radical scavenging capacity of 72.8 ± 2.9% and retarded thiobarbituric acid-reactive substances (TBARSs) formation in emulsified sausages stored at 4 °C for 7 days [146]. Niu et al. [147] found promising antioxidant activities from peptides (less than 1 kDa) obtained by fermentation of wheat germ with *Bacillus Subtilis* B1. They observed significant EC_50_ dose-dependent DPPH, hydroxyl and superoxide anion radical scavenging activities of 3.16 mg/mL, 6.04 mg/mL and 7.46 mg/mL, respectively. Studies by Wang et al. [148] showed that co-fermentation of barley with *Lactobacillus plantarum* and *Rhizopus oryzae* increased amino acid nitrogen, <10 kDa peptide, and free phenolic contents, and thus improved DPPH, hydroxyl, ABTS^+^ radical scavenging activity, and ferric reducing antioxidant power. Hydrolysates obtained during the fermentation of amaranth protein fractions with *Lactobacillus helveticus* and *Lactobacillus plantarum* possessed higher peroxyl and hydroxyl radical scavenging activities [149].

### 5.2. Bioinformatics Approach

Owing to the challenges involved in developing BP using classical approaches, which include time consumption, high cost, and uncertainties regarding the bioactivities of protein hydrolysates or fragment peptides which needs to be validated, bioinformatics could be a promising tool to discover bioactive peptides from different protein sources [150,151]. Bioinformatics, or in silico analysis, employs computational and statistical techniques to manage, curate, predict and interpret biological datasets [150]. Bioinformatics can minimize the number of experiments that must be performed to prepare BP by determining how their structure relates to their activity. Recently, researchers have employed the in silico approach to predict the production of BP from food proteins using bioinformatics and databases. This strategy, combined with classical approaches, can determine the optimum BP production parameters, such as the type of enzyme and target activity. In silico analysis has been used to predict peptides released by single and multiple enzyme digestion. Amino acid sequences and positions are key determinants of the bioactivities of peptides [152]. Several studies have attributed the bioactivies of peptides to the presence of some specific amino acids. For example, the amino acids cysteine (C), histidine (H), proline (P), methionine (M), and aromatic amino acids of food peptides have been reported to exert antioxidant activity [153]. Molecular docking approaches are used to predict and estimate the binding modes and affinities of small molecules within the binding sites of target receptors [154]. They have been used to screen for food-derived BP and illustrate their biological mechanisms. Protein structure selection and preparation, ligand preparation, docking, and analysis of results are the main steps involved in molecular docking [151]. In addition, bioinformatics approaches could be used to simulate and predict gastrointestinal stability, toxicity and allergenicity of peptides. Nonetheless, in vitro and in vivo validation of such a prediction needs to be carried out and ascertained. The bioavailability of antioxidant peptides is mainly affected by their transepithelial transport, and the human Caco-2 cell model is widely used for in vitro studies to investigate potential relevance in in vivo metabolism [135]. The in vivo challenges encountered by peptides as therapeutics has been comprehensively discussed by Yap and Gan [155]. Despite its wide use, increasing prediction accuracy of this computational tool can help overcome some theoretical and computational drawbacks.

Quantitative structure–activity relationship (QSAR) modeling reveals how the structural characteristics of compounds relates to their biochemical and functional properties. QSAR model development involves the following steps: (i) retrieving sequences of target peptides from a database or library; (ii) scalar description of amino acids constituents; (iii) QSAR model construction and activity prediction; and (iv) validation of synthesized peptides in vitro or in vivo. However, QSAR approaches are not without their limitations, as model development is difficult with lack of knowledge and the unavailability of protein sequences in protein libraries and online databases [156]. The reliability and predictability of a three-dimensional quantitative structure–activity relationship (3D-QSAR) model was developed using a combination of comparative molecular field analysis (CoMFA) and comparative similarity index analysis (CoMSIA), for a total of 198 antioxidant tripeptides retrieved from literature. Promising antioxidant activity was demonstrated from graphical contour maps of the model with significant contribution by electrostatic, steric, hydrophilic and hydrogen bond acceptor force fields. Consequently, ten novel tripeptides were designed with residue substitution, and their antioxidant activities were predicted by the model. Subsequently, the tripeptides were synthesized and validated by FRAP (ferric reducing antioxidant Potential) and ABTS (2,2′-azino-bis (3-ethlbenzthiazoline-6-sulfonic acid)) assays. Tripeptides WKW, GRC, ARW, LRW, LKW, and YKW showed higher ferric reducing capacity and ABTS radical scavenging capacity. Findings from this work showed a high correlation between experimental and predicted activity, and the developed model could provide insight regarding the structure and activity relationship of antioxidant peptides and be useful in their virtual screening and design [157]. Yan et al. [158] designed two novel tripeptides, GWY and QWY, using 3D-QSAR models, which demonstrated strong antioxidant activities of 3.32 mM TE and 2.97 mM TE, respectively; after synthesis and in vitro confirmatory evaluation using Trolox equivalent antioxidant capacity (TEAC) assay. These authors further investigated the potential molecular mechanism using molecular docking and molecular dynamics simulations. Their findings revealed that GWY and QWY could improve the body’s antioxidant defense system by competitively binding to Keap1′s active sites key residues Arg415, Arg483, Arg380 and Ser555, increasing the accumulation of Nrf2 and, hence, activating the Kelch-like ECH associated protein1 (Keap1)-nuclear factor erythroid 2-related factor 2 (Nrf2)-antioxidant response element (ARE) signaling pathway.

### 5.3. Emerging Food Processing Technologies

The potential application of innovative and emerging food processing techniques to improve food-derived protein digestibility, and produce BP of interest, is increasingly being explored (Table 5). HHP, a non-thermal and green technology which instantaneously and uniformly transmits isostatic pressure (100–1000 MPa) to enhance shelf life, improves the functional and bioactive properties of food products, and is an effective strategy to produce antioxidant peptides from various food sources. HHP treatment induces denaturation of native protein by disrupting hydrogen, as well as hydrophobic and ionic bonds, but not covalent and non-covalent bonds; hence, it modifies protein secondary structure but not the primary structure. Without the use of high temperatures, pressurization may improve the susceptibility of unfolded proteins access to enzyme hydrolysis [159,160]. Ultrasonication uses microbubble cavitation, and is considered an environmentally friendly food processing technique with higher yield, extraction rate, reproducibility, purity, minimal energy, water and solvent use. However, several factors, including ultrasound power, intensity, frequency, temperature, solvent, reactor design, as well as matrix parameters, are known to beneficially or negatively influence food components and metabolites [161]. Ultrasonication has been shown to increase protein extraction and sorghum digestibility. Sullivan et al. [162] found that ultrasonication at 40% amplitude for 10 min increased the solubility of sorghum kafirin protein from 6.5 μg/mL to 173.3 μg/mL, as well as its digestibility as a result of its secondary structure modification. In addition, ultrasonication followed by in vitro pepsin–pancreatin hydrolysis improved the antioxidant capacity of purified kafirin and sorghum gluten-like flour. Thus, ultrasonication could serve as a potential technique to improve the nutritional benefits and functionality of sorghum flour. Electron beam irradiation (EBI), an ionizing irradiation, is a safe, nonthermal, less expensive, and environmentally friendly technique used widely to modify food components and improve functional properties [163]. The functional and antioxidant properties of alcalase hydrolysates of wheat germ protein was remarkably improved after EBI treatment [164]. Li et al. [165] assessed the effect of EBI treatment on the structure and antioxidant activity of rice protein after alcalase hydrolysis. Even though EBI treatment induced amino acid oxidation as irradiation doses increased to 50 kGy, it caused changes in the secondary structure and hydrophobic regions in protein cores, leading to more fragmentations of hydrolysates and improvement in the antioxidant ability of rice protein. Irradiation at 50 kGy increased the DPPH and ABTS radical scavenging activity to 96.8% and 92.0%, respectively, compared to the 66.7% and 71.1% observed in non-irradiated rice protein hydrolysates.

## 6. Potential Mechanisms of Cereal Peptidic Antioxidants in MetS Prevention

Recent increase in MetS worldwide and its negative impact on quality of life has led to the search for food ingredients or materials with strong oxidative stress prevention properties. Owing to this, cereal antioxidant peptides have gained great attention among researchers. The amino acid composition, sequence and molecular weight are key factors that contribute to the antioxidant activities of peptides [80]. Due to the proteolytic actions of GI enzymes (pepsin, trypsin and chymotrypsin), BP may retain or lose their specific activities at targets sites upon oral ingestion. As such, in vitro activities may not necessarily translate into in vivo efficacies [155]. Over the years, the potential antioxidant mechanisms of peptides have mainly been investigated using in vitro chemical assays, in vitro cellular assays, and in vivo animal studies. Although in vivo mechanisms are not fully understood, the activation of the endogenous antioxidant defense system have been reported in vivo [135]. Radical scavenging properties, chelation of metal ions, and inhibition of lipid peroxidation assays are the in vitro chemical evaluation methods used to assess antioxidant capacities of peptides and other compounds. The two main mechanisms by which free radicals are deactivated in vitro are the hydrogen atom transfer (HAT) and single electron transfer (SET) [171]. The SET mechanism methods frequently used are the DPPH radical scavenging ability and the Trolox equivalent antioxidant capacity (TEAC), while oxygen radical absorption capacity (ORAC), total free radical capture antioxidant parameters (TRAP), and carotene bleach analysis utilizes the HAT mechanism. Ferric reducing antioxidant power (FRAP) and thiobarbituric acid (TBARS) are the most commonly used methods for evaluating metal ion chelation and lipid peroxidation inhibition of antioxidants, respectively [135]. Cell-based antioxidant evaluation assays provide a direct reflection of the cytoprotective abilities exerted on oxidation-induced damaged cells, compared to in vitro chemical methods (Table 6). Different human and non-human cell lines are commonly used to assess the antioxidant properties of peptides. Wang et al. [172] evaluated the underlying antioxidative mechanism of ADWGGPLPH, a wheat germ-derived peptide on hyperglycemia-induced oxidative stress in vascular smooth muscle cells (VSMCs). ADWGGPLPH significantly prevented cell proliferation induced by high glucose, reduced intracellular ROS production, and suppressed NOX4 protein expression (a key enzyme related to ROS production in vascular cells) via the PKCζ/AMPK signaling pathway. Furthermore, ADWGGPLPH treatment at 4 mg/kg, administered intraperitoneally, enhanced antioxidant abilities by reducing liver MDA levels and increasing SOD expression and attenuated inflammatory cytokine (plasma levels of TNF-α and IL-1β) generation in STZ-induced C57BL/6 diabetic mice. Thus, ADWGGPLPH as a dietary antioxidant supplement could be used to complement antihyperglycemic drugs in the treatment of diabetic vascular complications.

Although numerous studies have demonstrated the direct antioxidant actions of peptides in vitro using chemical assays, there is still limited understanding of their indirect activation of antioxidant and detoxifying molecular pathways in vivo (Table 7). Presently, the Keap1-Nrf2 signaling pathway is considered one of the plausible antioxidant mechanisms of peptides in vivo. Nuclear factor erythroid 2-related factor 2 (Nrf2) regulates cellular responses against environmental stresses, and is bound to Kelch-like ECH associated protein 1 (Keap1) in the cytoplasm under basal conditions. However, during oxidative stress conditions, Nrf2 is released from Keap1 and translocated into the nucleus, where it binds to antioxidant response elements (AREs) and upregulates target genes [173,174]. The indirect antioxidative effects of peptides have been shown to be mediated through the Keap1-Nrf2 pathway. The activation of Nrf2 protects cells from oxidative damage via the upregulation of antioxidant and detoxifying enzymes, such as heme oxygenase-1 (HO-1), glutathione reductase (GR), and NAD(P)H quinone oxidoreductase1 (NQO1). Oryza Peptide-P60 (OP60), a commercial rice peptide, was reported to increase intracellular glutathione levels. Moritani et al. [175] evaluated the mechanisms underlying the antioxidant potential of this peptide in HepG2 cells and mice models. Their results revealed the cytoprotective effect of OP60 via the Nrf2 signaling pathway. OP60 pretreatment of HepG2 cells at 5 mg/mL reduced cytotoxicity caused by H_2_O_2_ or acetaminophen (APAP). The mRNA level of genes encoding heavy and light subunits of γ-glutamylcysteine synthetase (γ-GCS) and other antioxidant enzymes were increased by OP60 treatment, as well as the promotion of Nrf2 nuclear translocation. OP60 treatment at 500 mg/kg in mice prevented oxidative stress-induced liver injury by increasing glutathione levels, heavy subunits of γ-GCS, and heme oxygenase-1 expression in the liver. Thus, OP60 could be utilized as a functional food or ingredient for the prevention or management of MetS associated with oxidative stress. Findings from Sullivan et al. [176] revealed an association between the anti-inflammatory potential of sorghum kafirin in LPS-induced inflammation in THP-1 cells, and a reduction of intracellular ROS production. This association may be due to the possibility of kafirin’s ability to directly bind to LPS and inhibit LPS binding to its receptor, TLR4, in kafirin-treated THP-1 cells, thus reducing ROS production induced by LPS. Moreover, reduced secretion of pro-inflammatory cytokines (IL-1β, IL-6 and TNF-α) could be due to decreased nuclear translocation of p65 NF-κB subunits and c-JUN, as a result of limited phosphorylation of ERK 1/2 and JNK caused by a reduction in intracellular ROS production.

The inhibition of Toll-like receptor 4 (TLR4) pathways and the suppression of NF-κB activation is one of the potential mechanisms by which antioxidative peptides exert their protective functions. The antioxidant effect of rice-derived bran bioactive peptides (RBAP), KHNRGDEF, on H_2_O_2_-induced oxidative injury in human umbilical vein endothelial cells (HUVECs) was evaluated by Liang et al. [177]. RBAP protected HUVECs against H_2_O_2_-induced oxidant injury by binding to, and inhibiting, Toll-like receptor 4 (TLR4) pathways and suppressing NF-κB activation. Cruz-Chamorro et al. [178] reported on the reduction of Type 1 T helper (Th1) and Th17 pro-inflammatory cytokines production, suggesting an improvement in cellular anti-inflammatory microenvironment by increasing Th2/Th1 and Th2/Th17 balances in phytohaemagglutinin-P (PHA)-stimulated human peripheral blood mononuclear cells (PBMCs) using alcalase-generated wheat germ protein hydrolysates (WGPHs) treatment. WGPHs improved the total antioxidant capacity by directly scavenging free radicals, increasing the reduced form of glutathione (GSH) levels, and reducing nitric oxide (NO) overproduction. The antioxidative and anti-inflammatory effects of three peptides (MPH-A-I, IALLIPF, and PFLF) from foxtail millet, obtained by alcalase hydrolysis, was evaluated in H_2_O_2_-induced human keratinocyte HaCaT cells and RAW264.7 murine macrophages [179]. Peptides (IALLIPF and PFLF) from millet prolamins demonstrated promising antioxidant activity by effectively reducing ROS production in H_2_O_2_-induced HaCaT cells, inhibiting MDA production, and increasing GSH levels more than the peptide fraction of MPH-A-I. Furthermore, peptides suppressed the production of NO and pro-inflammatory cytokines (TNF-α, IL-1β and IL-6) in (lipopolysaccharide) LPS-stimulated RAW264.7 cells. In addition, pretreatment with millet peptides was found to suppress the production of phosphorylated proteins (P-p38, P-Erk 1/2, and P-JNK) in the MAPK pathway. The potential antioxidant and anticancer activities of papain–hydrolyzed sorghum kafirin peptide antioxidants in human hepatocarcinoma (HepG2) cells have been demonstrated by Xu et al. [132]. They found that after 72 h treatment, antioxidant peptides (1–3 kDa) at 50 and 200 μg/mL significantly inhibited the growth of HepG2 cells, without any negative effect on cell viability. Findings from Wang et al. [180] showed intracellular ROS scavenging activities and the regulation of antioxidant defense and ROS metabolism relevant gene expressions in HepG2 cells by alcalase-derived corn gluten hydrolysate (CGH). Pretreatment of cells with CGH1 enhanced the expression of several genes (GPX3, GPX5, SOD3, CYGB, SEPP1, and MT3) initially suppressed in H_2_O_2_-induced HepG2 oxidative damaged cells. The suppression of EPHX2 expression by CGH1, which could be attributed to its antioxidant effect, was associated with arachidonic acid metabolism shown by an increase in cellular epoxyeicosatrienoic acid (EET) and EET-phospholipids formation. Miscalculation and misinterpretation of effective animal doses are two of the main problems encountered in interspecies comparisons and safe starting dose determination for initial clinical trials. Reagan-Shaw et al. [181] proposed the use of a body surface area (BSA) normalization method as an appropriate means for translating doses from animal studies to human equivalent doses (HEDs); instead of using a simple conversion based on body weight, which oftentimes results in misinterpretation and inappropriate comparisons between species due to invalid or inaccurate calculations. The authors suggested BSA as a suitable conversion factor for clinical trials, as it correlates well with several mammalian biological parameters, such as oxygen utilization, basal metabolism, caloric expenditure, blood volume, circulating plasma proteins, and renal function. However, for an average 60 kg person, an estimated dose of 3891.6 mg, 2432.4 mg, and 972 mg is required for corn germ albumin peptide fraction 4, OP60 rice commercial peptide, corn gluten meal peptides, and wheat bran peptides HEDs of 64.86, 40.54, 16.22 mg/kg, respectively (Table 7). These concentrations are not reasonably obtainable compared to a dose of 19.2 mg and 447.60 mg for wheat bran peptides, ADWGGPLPH and LRP, with an HED of 0.32 and 7.46 mg/kg, respectively. In addition, the size of the peptide, the mode of administration—whether oral, injection or perfusion—and the delivery system have a great impact on peptide stability, transport, and bioavailability.

## 7. Conclusions

Cereals are important staple foods which account for a huge proportion of the human diet. Recent human and animal studies have revealed compelling evidence of the association between oxidative stress and MetS. This review highlights recent technological advancements in the production of antioxidant peptides from cereals, although large scale applicability is still lacking and is required in the future. The simultaneous application of green technologies, such as high hydrostatic pressure, ultrasonication, and electron beam irradiation with enzymatic and fermentation methods, can help improve protein digestibility and produce mono- and multifunctional peptides from cereals and their byproducts. The potential of cereal protein hydrolysate and peptides to prevent oxidative stress in vitro, using chemical and cellular-based assays, has been well documented in existing literature. However, in vivo evaluations in animals are still few, and they are also lacking in human studies. As such, future studies are needed to elucidate the potential of peptide antioxidants and their intestinal absorption mechanisms under physiological conditions. In addition, future clinical studies to establish optimum safe and efficacious doses are needed. In summary, cereal peptidic antioxidants have great potential to be developed as health-promoting functional ingredients, foods, or dietary supplements for the prevention and management of MetS.

## Figures and Tables

**Table 1:** Some proposed definitions of oxidative stress throughout the course of time.

Definition of Oxidative Stress	Reference
Oxidative stress is a disturbance in the prooxidant–antioxidant balance in favor of the former.	[24]
Oxidative stress is defined as a disturbance in the prooxidant–antioxidant balance that leads to potential damage.	[25]
Oxidative stress is a situation when steady-state ROS concentration is transiently or chronically enhanced, disturbing cellular metabolism, its regulation, and damaging cellular constituents.	[20]
Oxidative stress is defined as excess production of reactive oxygen species (ROS) relative to antioxidant defense.	[26]
Oxidative stress refers to the imbalance between cellular antioxidant cascade and processes that generate reactive oxygen species (ROS), such as superoxide (O_2_^.^), hydrogen peroxide (H_2_O_2_), and hydroxyl anion (OH^−^).	[27]
Oxidative stress is defined as an imbalance between oxidants and antioxidants in favor of the oxidants, leading to a disruption of redox signaling and control and/or molecular damage. This implies a deviation to the opposite side of the balance (thus, “reductive stress”), as well as physiological and supraphysiological deviations, which tie into the overarching concept of “oxidative distress” and “oxidative eustress”, respectively.	[5]

**Table 2:** Types/Levels of oxidative stress.

Oxidative Stress	Definition	Reference
Acute	The inability of cells to neutralize enhanced ROS level over a period of time, such that the time is enough to result in specific physiological consequences.	[20,21,22,23]
Chronic	It occurs when acute oxidative stress progresses to significantly disturb homeostasis. Here, the ROS levels are elevated and very stable, and very potent in altering healthy cell components.	[20]
Quasi-stationery	It occurs when ROS levels are so elevated that it is almost impossible to return to ideal homeostatic levels, thus resulting in the need for a substantial reorganization of the entire homeostatic system.	[22,23]

**Table 3:** Major assortments of cereals-specific genus.

Cereal	Family	Sub-Family	Tribe	Genus	Reference
Wheat	Gramineae or Poaccac	*Pooideae Bentham*	Triticeae	Triticale	[49,56]
Rye		*Pooideae Bentham*	Triticinae	Triticale	[49,56]
Barley		*Pooideae* Bentham	Hordeinae	Triticale	[49,56]
Oat		*Pooideae* Bentham	Aveneae	Aveneae	[56]
Millet		*Panicoideae* Link	Paniceae	Panicease	[49]
Sorghum		*Panicoideae* Link	Andropogoneae	Sorghum	[49]
Maize		*Panicoideae* Link	Maydeae	Zea	[49,56]
Rice		*Ehrhartoideae* Link	Oryzeae	Oryza	[49,56]

**Table 4:** Chemical assay showing some examples of cereal antioxidant peptides.

Protein Source	Enzyme	Peptide Sequence/Hydrolysate	Size	Assay Outcome	Reference
Wheat bran	Alcalase	NL, QL, FL, HAL, AAVL, AKTVF, and TPLTR	<1, 1–10 kDa, (membrane ultrafiltration)	Higher ORAC by <1 kDa fraction.	[108]
Wheat bran	Alcalase, favourzyme, papain, neutral enzyme, trypsin	CGFPGHC, QAC, RNF, SSC, WF	<1 kDa, (size-exclusion and and ion-exchange chromatography)	QAC and SSC from alcalase hydrolysate showed the highest superoxide anion radical scavenging and Trolox equivalent antioxidant capacity.	[109]
Wheat germ	Alcalase, pepsin or proteinase K	KELPPSDADW, SGGSYADELVSTAK, GNPIPREPGQVPAY	<2.5 kDa, (RP-HPLC)	Pepsin fraction showed the strongest ABTS and DPPH radical scavenging activity.	[110]
Wheat gluten	Pepsin	LQPGQGQQG, AQIPQQ	<5 kDa, (ion-exchange and gel filtration chromatography)	Fraction 3 showed stronger antioxidant activity using ferric thiocyanate method.	[111]
Wheat germ	proteinase K	SGGSYADELVSTAK, MDATALHYENQK	<3 kDa, (RP-HPLC)	This fraction displayed strong ABTS and DPPH radical scavenging activity.	[112]
Wheat gluten	Alcalase	Hydrolysate	Not determined	Heat and alcalase treated hydrolysate synergistically increased DPPH and ABTS radical scavenging inhibition.	[113]
Rice bran	Alcalase, Trypsin, Protamex, Flavourzyme	Hydrolysate	N.R	Flavourzyme hydrolysate exhibited the strongest DPPH, ABTS radical scavenging and metal chelating activity.	[114]
Rice bran	Trypsin	YSK	<1 kDa, (gel filtration, RP-HPLC)	F2-a fraction displayed strong DPPH radical scavenging and reducing power activity	[115]
Brown rice	Bromelain	GSGVGGAK, SSVGGGSAG, FGGSGGPGG, FGGGGAGAGG, GGGGGAAAAGA, AGGGGGGVVAG, SGPSGGGGAL, ESDVVSDL	<1 kDa, (ultra, gel filtration, RP-HPLC)	DPPH, ABTS, and hydroxyl radical-scavenging activities was higher in F4 fraction.	[69]
Brown rice	Bromelain	SPFWNINAHS, MPVDVIANAYR, VVYFDQTQAQA, AVYVYDVNNNANQ, YNILSGFDTEL, EFFDVSNELFQ	<1.5 kDa, (ultra, gel filtration, RP-HPLC)	F4 fraction of glutelin hydrolysates showed the highest ABTS radical scavenging and Cu^2+^ chelating activity.	[116]
Rice bran	Pepsin, Trypsin	N.R	3–10 kDa, (ultra, gel filtration, RP-HPLC)	F2 sub-fraction displayed the highest DPPH, ABTS radical scavenging and Fe^2+^ chelating activity.	[117]
White and colored rice	Alcalase, Flavourzyme, Neutrase	Hydrolysate	<10 kDa	Alcalase hydrolysate of immature rice showed the highest DPPH and Fe^2+^ chelating activity compared to matured rice.	[118]
Rice	Pepsin-Pancreatin	Hydrolysate	N.R	Methionine-supplemented hydrolysates showed enhanced scavenging capacities for ABTS, superoxide, and nitric oxide.	[119]
Rice bran	pepsin-trypsin	N.R	<3 kDa, ultrafiltration	Higher ABTS radical scavenging and metal chelating activities in F1 fragment.	[120]
Rice	Alcalase, Flavourzyme	Hydrolysate	<3 kDa	Alcalase hydrolysate showed higher DPPH and FRAP activities and, hence, improved oxidative stability of linseed oil during storage.	[121]
Rice	Microbial proteases	Hydrolysate	1–3 kDa, ultrafiltration	Validase and alkaline protease F3 fractions exerted remarkable DPPH and ABTS radical scavenging abilities. Validase-F3	[122]
	(Validase^®^, Neutral Protease, alkaline protease).			(500 µg/g) inhibited lipid oxidation by 19 and 15% at days 8 and 15 of storage, respectively.	
Rice residue protein	Alcalase, Flavourzyme, Protamex, Pepsin, Trypsin, Papain	RPNYTDA, TSQLLSDQ, TRTGDPFF, NFHPQ	<1 kDa, ultrafiltration, SEC, RP-FPLC	RRPB3 fraction showed the strongest DPPH, ABTS radical scavenging and FRAP-Fe^3+^ reducing activities. Synergistic activity was displayed by RRPB3 I and III.	[123]
Corn gluten meal	Neutrase	Hydrolysate	-	Hydrolysate (5 mg mL^−1^) inhibited lipid oxidation in emulsion by reducing hydroperoxides and TBARS formation.	[124]
Corn germ meal	Alcalase	MGGN, MNN, MEN	<1 kDa, (ultra, gel filtration)	F3 fraction from hydrolysate-5 displayed higher DPPH, ABTS radical scavenging, and ORAC activities.	[125]
Corn gluten meal and distillers’- dried grains with solubles (DDGS)	Neutrase,	Hydrolysate	-	Neutrase-hydrolyzed corn gluten meal (2%) displayed the strongest oxidation prevention in ground meat and significantly reduced TBARS in pig feed. Alcalase-hydrolyzed DDGS retarded the lipid oxidation by 37.8%.	[126]
	Alcalase				
Corn protein isolate	Microbial protease (Validase, Alkaline, Neutral)	Hydrolysate	<1–10 kDa, (ultrafiltration)	Neutral protease fraction (NP-F3) demonstrated the highest ORAC peroxyl radicals scavenging activity. NP-F3 (250 and 500 µg/g) remarkably inhibited lipid oxidation by 52.9% in ground beef.	[127]
Corn gluten meal	Alcalase, Protamex	QQPQPW	<1 kDa (ultra, gel filtration and RP-HPLC).	Alcalase hydrolysate displayed the highest antioxidant activity. Hexapeptide showed remarkable DPPH, ABTS, and hydroxyl radical scavenging activity, but weaker Fe^2+^-chelating capacity.	[128]
Corn gluten meal	Alcalase,	YPQ, AYL	<1 kDa (ultra, gel filtration and RP-HPLC)	Alcalase hydrolysates and GI-resistant peptides exhibited higher DPPH, hydroxyl radical scavenging and reduced power activity.	[129]
	Pepsin, pancreatin				
Corn gluten meal	Papain, Ficin, Bromelain	Hydrolysate	<1 kDa, >10 kDa (ultra, gel filtration)	Papain F4 fraction, ficin F1 fraction, and bromelain F3 fraction showed the strongest DPPH and ABTS radical scavenging and metal chelating activity. Papain F4 fraction (1000 mg/kg) reduced lipid oxidation in ground meat by 30.45%.	[130]
Corn gluten meal	Alcalase, Flavourzyme, Alcalase + Flavourzyme and Flavourzyme + Alcalase	CSQAPLA, YPKLAPN, YPQLLPNE	<1 kDa (ultra, gel filtration and RP-HPLC	Alcalase + Flavourzyme hydrolysate exhibited better antioxidant activities. CSQAPLA displayed the highest DPPH and superoxide anion radical scavenging capacities, and good reducing power activity.	[96]
Sorghum	Alcalase	VAITLTMK, VSKSVLVK	<1 kDa, (ultra, gel filtration, RP-UFLC)	These peptides displayed the highest DPPH radical scavenging activity.	[102]
Sorghum	Alcalase	QQWQ, QWQQ	<5–10 kDa, (ultra, gel filtration)	Medium-sized hydrolysate fraction exhibited stronger antioxidant activities based on DPPH, ABTS, metal chelating, and reducing power assay. The fraction also exerted remarkable lipid oxidation inhibition, both in emulsion and in the ground meat system.	[131]
Finger millet	Trypsin, pepsin	TSSSLNMAVRGGLTR, STTVGLGISMRSASVR	<3 kDa, (ultra, gel filtration and RP-UFLC)	Trypsin fraction GFB exhibited the highest DPPH and ABTS radical scavenging activity. In silico interaction of serine and threonine residues with free radicals potentially resulted in antioxidant activity.	[101]
Sorghum	Papain	LRQQ, QLQGV, WQPN, GLQDL, AMCGVV, YLRQ, TPCATS, QGVAAA, AQVAQ, QQLQ	1–3 kDa (ultrafiltration and gel filtration)	Kafirin hydrolysates (1.0 mg/g) inhibited TBARS formation by 32.1% in meat samples. Fraction F3 also exhibited the highest % of DPPH radical scavenging activity, metal chelating and ORAC activity.	[132]
Sorghum	Alcalase, flavourzyme, neutrase, everlase, protamex, papain, ficin, bromelain, trypsin, Pepsin	Neutrase hydrolysate fractions	3–10 kDa (ultra and gel filtration)	Neutrase F2 hydrolysates fraction displayed higher antioxidant activities (DPPH, ORAC, metal chelating) and retarded lipid autoxidation and peroxidation in the meat model.	[133]

ORAC: oxygen radical absorbance capacity; GI: gastrointestinal; TBARS: thiobarbituric acid reactive substances; N.R: not reported.

**Table 5:** Cereal antioxidant peptides using different processing methods.

Protein Source	Processing Method	Peptide Sequence/Size/Hydrolysate	Assay Outcome	Reference
Wheat germ protein hydrolysates	Electron beam irradiation (EBI)	<1 kDa	Irradiation at 50 kGy increased DPPH and ABTS^+^ radical scavenging activity by 45.77% and 52.52%, respectively.	[164]
Wheat germ	Fermentation with *Bacillus subtilis*	N.R	Peptide content increased in fermented samples compared to non-fermented samples and resulted in an increase in DPPH radical scavenging, Fe^2+^ chelating and Fe^3+^ reducing power activities.	[166]
KAMUT^®^ Wheat	Combination of enzyme hydrolysis (Alcalase, Neutrase, Flavourzyme) and fermentation with (Lactobacillus spp. strains)	VLPPQQQY	Stronger superoxide anion, hydroxyl radicals, organic nitro-radicals (ABTS, DPPH) scavenging, and lipid peroxidation inhibition was observed.	[167]
Wheat germ	Subcritical water extraction	GPFGPE, FGE, <1 kDa	Peptide fraction 4 showed the strongest DPPH radical scavenging activity and could effectively cross Caco-2 intestinal epithelium cells.	[168]
Sorghum	Ultrasonication combined with pepsin-pancreatin hydrolysis	<1 kDa	Ultrasonication increased DH, DPPH scavenging activity and ORAC values. However, there was no improvement in NO scavenging activity.	[162]
Rice dregs	Angling method using metal-organic framework combined with alcalase hydrolysis.	GDMNP, LLLRW	Strong DPPH, superoxide anion, hydroxyl radical scavenging and Fe^2+^ chelating activity was exhibited by peptides.	[169]
Rice	Alcalase-assisted electron beam irradiation (EBI)	Hydrolysate	EBI treatment at 50 kGy improved DPPH (96.81%) and ABTS (92.04%) radical scavenging activity.	[165]
Corn gluten meal	Ultrasonication assisted alcalase hydrolysis	SGV, LPF, LLPH, LLPF, FLPF, AHL, LGV (<1 kDa)	Ultrasonic pretreatment (5 W/L, 2 s/2 s on/off, 50 °C, and 25 min) significantly increased DH, DPPH and hydroxyl radical scavenging activity and enhanced formation of small size peptides.	[170]

**Table 6:** Cellular antioxidant effects of cereal peptides and hydrolysates.

Protein Source	Enzyme	Peptide Sequence/Hydrolysate	Cellular Model	Cellular Outcome	Reference
Wheat germ	Trypsin, Alcalase	AREGETVVPG	Vascular smooth muscle cells	High glucose-induced cell growth and generation of intracellular ROS was significantly decreased by AOP (5 µM). Suppression of PKCζ, AKT and Erk1/2 phosphorylation, and inhibition of Nox4 protein expression by AOP (5 µM).	[182]
Wheat germ	neutral protease	ADWGGPLPH	Vascular smooth muscle cells	High glucose-induced cell proliferation and intracellular ROS generation was significantly reduced by peptide at 10 µM and 20 µM. Stimulation of AMPK activity, inhibition of PKCζ, AKT and Erk1/2 phosphorylation, and suppression of NOX4 protein expression.	[172]
Wheat gluten protein	Alcalase	Protein hydrolysate	Human peripheral blood mononuclear cells	Hydrolysate (0.5 mg/mL) directly scavenged free radicals, increased GSH levels, reduced NO overproduction, and, thus, enhanced cells’ antioxidant capacity. Also, cell proliferation, Th1 and Th17 pro-inflammatory cytokines IFN-γ and and IL-17 were reduced.	[178]
Foxtail millet	Alcalase	PFLF, IALLIPF	Human keratinocyte HaCaT cells	ROS, MDA production was effectively reduced and GSH levels increased by MPP (300 µg/mL) in H_2_O_2_-induced HaCaT cells.	[179]
Sorghum	Pepsin-pancreatin	Kafirin hydrolysate	THP-1 human macrophages	Kafirin (100 μg/mL) reduced LPS-induced intracellular ROS production, inflammatory cytokines (IL-1β, IL-6 and TNF-α) production, and nuclear translocation of p65 and c-JUN.	[176]
Rice bran	-	KHNRGDEF	Human umbilical vein endothelial cells (HUVECs)	H_2_O_2_-induced HUVECs oxidant injury was protected by rice bran peptide (0.1 mM) supplementation via TLR4 binding, pathway inhibition, and suppression of NF-κB activation.	[177]
Rice	-	OP60 commercial peptide	HepG2 cells	H_2_O_2-_ or APAP-induced HepG2 cytotoxicity was reduced by 5 mg/mL OP60 pretreatment via glutathione homeostasis restoration and increased mRNA expression of antioxidant enzymes.	[175]
Corn	Alcalase	Zein hydrolysate/peptides	HepG2 cells	Hydrolysate showed higher ORAC activity than native proteins. Peptides (1155.56–1781.63 ng/mL IC_50_) induced apoptosis at 24 hr by increasing caspase 3 expression.	[183]
Corn germ meal	Alcalase	MGGN, MNN, MEN	HepG2 cells	Peptides (0.2 mM) significantly reduced ROS generation in H_2_O_2_-induced HepG2 cells. MNN showed the highest cellular antioxidant activity.	[125]
Corn gluten meal	Alcalase	corn gluten hydrolysate (CGH1) <1 kDa	HepG2 cells	CGH1-pretreated cells at 2.5 mg/mL upregulated the genes GPX3, GPX5, SOD3, CYGB, SEPP1, and MT3 involved in antioxidant defense. CGH1 suppressed EPHX2 expression, increased cellular EETs, EET-phospholipids formation, and, thus, protected against H_2_O_2_-induced HepG2 cell damage.	[180]
Corn gluten meal	Alcalase	GLLLPH	HepG2 cells	Corn peptide fractions (CPF) at 2.50 mg/mL exhibited high cellular antioxidant activities and increased the levels of intracellular antioxidant enzymes (SOD, CAT, GR and GSH) in oxidized HepG2 cells.	[73]

ROS: reactive oxygen species; AOP: antioxidant peptide; NOX: NADPH oxidase; PKCζ: phospho-protein kinase ζ; AMPK: AMP-activated protein kinase; Erk: extracellular signal–related kinase; AKT: Th1: Type 1 T helper; Th17: Type 17 T helper; IFN-γ: interferon-γ; IL17: interleukin 17; GSH: reduced glutathione; GR: glutathione reductase; SOD: superoxide dismutase; CAT: catalase; NO: nitric oxide; MDA: malondialdehyde; MPP: millet prolamins peptides; LPS: lipopolysaccharide; TLR4: Toll-like receptor 4; EET: epoxyeicosatrienoic acid.

**Table 7:** Antioxidant effects of cereal-derived peptides and hydrolysates in animal models.

Protein Source	Peptide/Hydrolysate	Purpose of Study	In Vivo Outcome	BSA-Based Human Equivalent Dose (mg/kg)	Reference
Wheat bran	NL, QL, FL, HAL, AAVL, AKTVF, and TPLTR	Investigated the blood pressure lowering effects of wheat protein hydrolysates and peptides in spontaneous hypertensive rats (SHR).	Oral administration of <1 kDa peptides at 100 mg/kg showed a better reduction of systolic blood pressure (−35 mmHg) after 6 h compared to hydrolysate (−20 mmHg).	16.22	[108]
Wheat bran	LRP, LQP	Evaluated the effects of peptides on oxidative stress and the AMPK signaling pathway in HFD-induced NASH C57BL/6 mice.	NASH mice treated with 0.20% LRP showed a remarkable decrease in serum d-ROM and a significant increase in BAP levels. Administration of 0.20% LQP increased phospho-AMPK expression and decreased phospho-ACC expression, thus alleviating the severity of NASH.	7.46	[184]
Wheat bran	ADWGGPLPH	Assessed the antioxidative and antidiabetic vascular dysfunction effects of peptide in STZ-induced C57BL/6 mice.	Administered peptide at 4mg/kg enhanced SOD expression and total antioxidant capacity, and also attenuated hyperglycemia-induced inflammatory factors, such as TNF-α and IL-1β.	0.32	[172]
Rice	OP60 commercial peptide	Evaluated the protective effect of OP60 against APAP-induced hepatic injury in mice.	GSH synthesis and antioxidant enzymes were induced by OP60 (500 mg/kg) via activation of the Nrf2 pathway.	40.54	[175]
Corn Gluten meal (CGM)	CGM peptides (<10 kDa)	Investigated the antioxidant capacity of CGMP produced by solid–state fermentation with *Bacillus subtilis* MTCC5480 in aging rats induced with D-galactose.	Serum and liver antioxidant enzymes (SOD, catalase, glutathione peroxidase) and total antioxidant capacity activities increased, with a decrease in MDA in D-galactose-induced aging rats fed CGMP (250 mg/kg bw).	40.54	[185]
Corn germ meal	Albumin peptides fractions (APF-4)	Examined the hepatoprotective effects of APF-4 in alcohol-induced liver injury in mice.	APF-4 (800 mg/kg bw) administration significantly reduced activities and levels of hepatic (AST), ALT and MDA, and increased activities of SOD, CAT and GSH.	64.86	[186]

HFD: high-fat diet; AMPK: AMP-activated protein kinase; NASH: non-alcoholic steatohepatitis; d-ROM: diacron reactive oxygen metabolite; BAP: biological antioxidant potential; ACC: acetyl-CoA carboxylase; STZ: streptozotocin; TNF-α: tumor necrosis factor α; IL-1β: interleukin-1β; Nrf2: nuclear factor erythroid 2-related factor 2; acetaminophen: APAP; SOD: superoxide dismutase; CAT: catalase; GSH-Px: glutathione peroxidase; MDA: malondialdehyde, BSA: body surface area. Human equivalent dose (HED) based on BSA was calculated according to the formula suggested by Reagan-Shaw et al. [181].
